# Diffuse correlation spectroscopy: current status and future outlook

**DOI:** 10.1117/1.NPh.10.1.013509

**Published:** 2023-01-24

**Authors:** Stefan A. Carp, Mitchell B. Robinson, Maria A. Franceschini

**Affiliations:** Massachusetts General Hospital, Harvard Medical School, Optics at Martinos Research Group, Charlestown, Massachusetts, United States

**Keywords:** diffuse correlation spectroscopy, multispeckle detection, interferometric detection, pathlength-resolved detection, near-infrared, blood flow

## Abstract

Diffuse correlation spectroscopy (DCS) has emerged as a versatile, noninvasive method for deep tissue perfusion assessment using near-infrared light. A broad class of applications is being pursued in neuromonitoring and beyond. However, technical limitations of the technology as originally implemented remain as barriers to wider adoption. A wide variety of approaches to improve measurement performance and reduce cost are being explored; these include interferometric methods, camera-based multispeckle detection, and long path photon selection for improved depth sensitivity. We review here the current status of DCS technology and summarize future development directions and the challenges that remain on the path to widespread adoption.

## 1 Introduction

Diffuse correlation spectroscopy (DCS) has emerged over the last decade as a versatile technique for noninvasive tissue perfusion measurements using near-infrared light.^1^^,^^2^ As an extension of the dynamic light scattering technique^3^ to multiply scattered light in tissue, DCS quantifies blood flow from the fluctuations in the intensity of diffusely scattered coherent light. The fluctuations result from the changing interference pattern at the detector due to moving tissue scatterers, a phenomenon primarily driven by red blood cell (RBC) movement.^4^

Typical DCS implementations use a long-coherence length laser for illumination and single mode fibers coupled to photon counting detectors to sample the intensity fluctuations of individual speckles on the tissue surface. The photon detection signals are then routed either to a hardware correlator or to a time-tagger that then streams the photon detection timestamps for postprocessing in the control computer using software autocorrelation algorithms. The fundamental DCS measurement is the normalized temporal intensity auto-correlation function $g_{2}(\tau )\equiv \frac{\left\langle I(t)I(t+\tau )\right\rangle }{{\left\langle I(t)\right\rangle }^{2}}$, where I(t) is the measured light intensity and τ is the correlation lag time. In the majority of the work in the field so far, an analytical model based on the correlation diffusion equation^5^ is then used to fit the measured $g_{2}$ and extract a blood flow index (${\mathrm{BF}}_{i}$). A key element in this process is choosing a motion model to link ${\mathrm{BF}}_{i}$ with the scatterer mean square displacement: diffusion (random walk), $\langle \Delta r^{2}(\tau )\rangle =6{\mathrm{BF}}_{i}\tau$, or convection (random flow), $\langle \Delta r^{2}(\tau )\rangle ={\mathrm{BF}}_{i}^{2}\tau^{2}$. Despite the apparent convective flow nature of RBC motion in vasculature, substantial experimental evidence indicates that a diffusive motion assumption for scatterer mean square displacement is needed for a good match of the DCS theoretical model with experimental data.^2^ The shear-induced diffusion process^6^ was proposed as an explanation, and simulations indicate that diffusive motion likely dominates DCS recordings under typical experimental conditions.^7^ However, sensitivity to convective motion could be seen under certain conditions.^7^^–^^10^

**Fig. 1:**
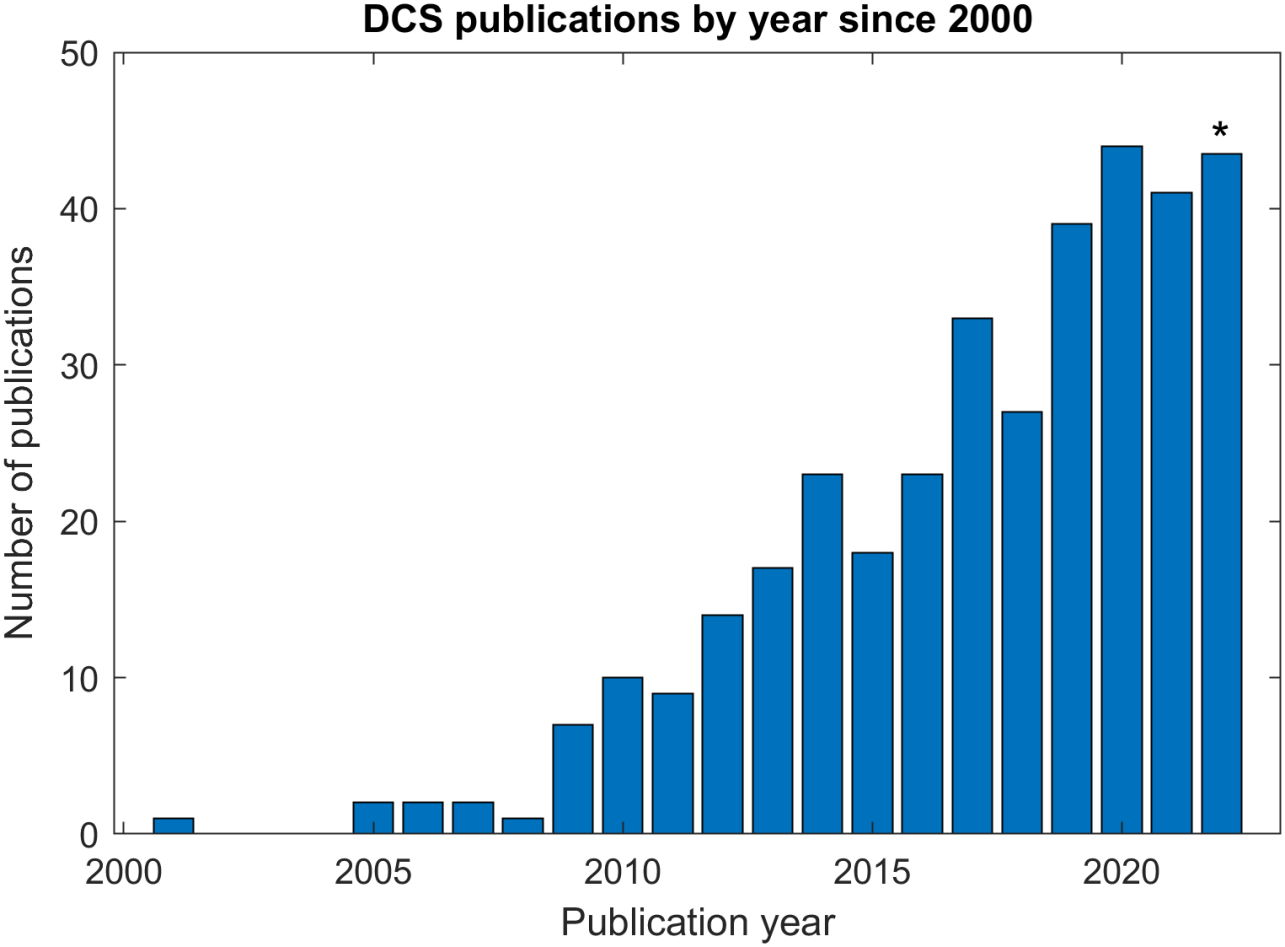
Number of papers mentioning DCS in their title or abstract based on a PUBMED search (*value for 2022 extrapolated as of the date of writing).

Notwithstanding the remaining open questions on the origins of the DCS “signal,” ${\mathrm{BF}}_{i}$ has been shown to be reliably proportional to tissue blood flow through validation against a number of “gold-standard” techniques, including arterial spin-labeling magnetic resonance imaging (ASL-MRI),^11^^–^^13^ fluorescent microspheres,^14^ transcranial Doppler ultrasound (TCD),^15^^,^^16^ xenon-enhanced computed tomography (Xe-CT),^17^ bolus tracking time-domain near-infrared spectroscopy (NIRS),^18^^,^^19^ phase-encoded velocity mapping MRI,^20^ and O215 positron emission tomography (PET).^21^ Encouraged by these validation studies, a wide range of potential applications, primarily in neuromonitoring (see Refs. 1 and 22 for comprehensive reviews), but also in breast cancer,^23^^,^^24^ muscle physiology^25^^–^^27^ and animal models of diverse pathologies,^28^^–^^33^ have been demonstrated. As seen in Fig. 1, the field has steadily grown over the past 15 years with more than 350 publications to date (this is an underestimate as some papers have been published under the name of “diffusing wave spectroscopy,”^34^ the predicate technique for DCS in the soft matter physics field).

## 2 Benefits and Challenges

In addition to its intrinsic value as a noninvasive deep tissue perfusion monitoring method, DCS has several other beneficial characteristics. One is the simplicity of the hardware, consisting of just a few (admittedly expensive) components and a fully digital signal processing chain with no calibration or gain adjustments required. Further, because tissue driven intensity fluctuations are generally above 100 Hz, slow light intensity changes do not impact the recorded autocorrelations. As such, ${\mathrm{BF}}_{i}$ tends to return to the previous level after a motion artifact as long as overall contact is not lost, whereas a purely intensity-based measurement, as used in NIRS, for example, might show a significant step change in signal level.

Nevertheless, DCS in its standard implementation suffers from several significant challenges, some shared with NIRS (limited depth penetration, sensitivity to crosstalk from superficial physiology) and some specific to DCS (signal to noise ratio (SNR) limitations, difficulty in interpreting absolute ${\mathrm{BF}}_{i}$ values). Although the discussion below focuses on cerebral blood flow (CBF) monitoring, as the primary application area for DCS in the field, considerations of depth sensitivity and SNR are relevant to the full range of potential DCS applications.

**Fig. 2:**
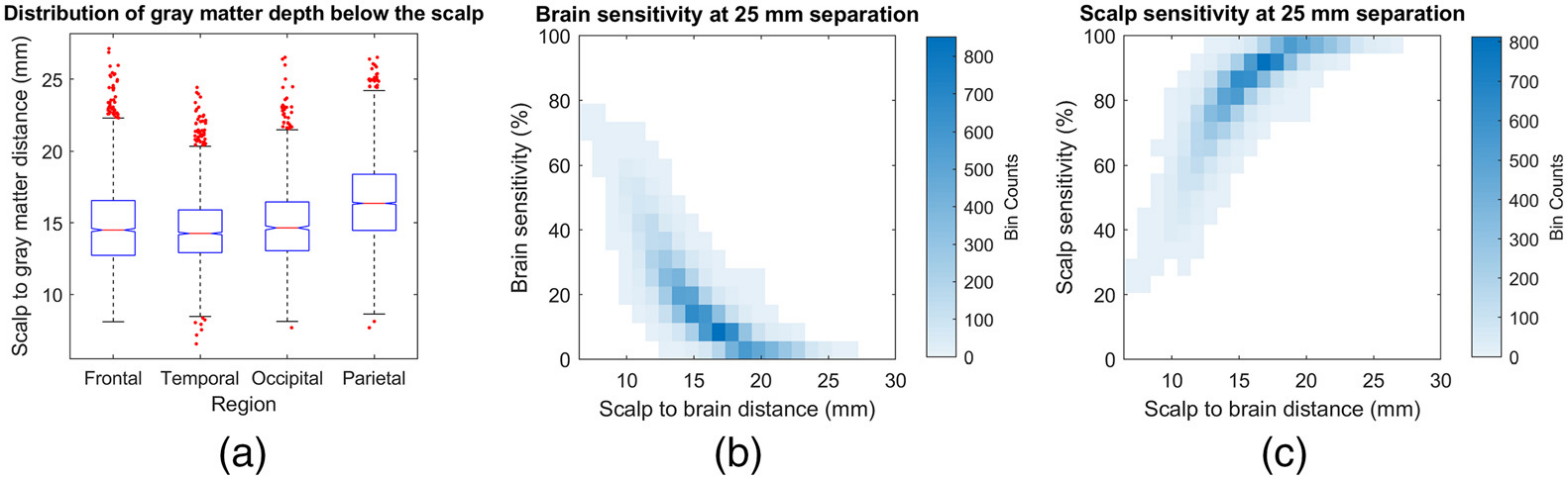
Brain sensitivity evaluation for DCS at 25 mm source-detector separation. (a) Boxplot of distance between scalp and gray matter surface in different areas of the head (first, second (median), and third quartile range shown, with outliers defined as more than 2.67 standard deviations) from segmented MRI scans; (b) and (c) fraction of true change recovered from brain and superficial (scalp) tissue, respectively, as a function of the local distance between the scalp and gray matter surfaces.

To illustrate, Fig. 2(a) shows the distribution of the straight line distance between the scalp surface and gray matter in different areas of the head, derived from a set of segmented MRI scans collected as part of a previous study (16 subjects, average age 29, range 25 to 41),^35^ and subplots shown in Figs. 2(b) and 2(c) show the fractional recovery of a true blood flow change in the scalp and brain tissue, respectively, using simulation data from the same study^35^ for the 25-mm source-detector separation used in the majority of published DCS investigations. As can be seen in these graphs, not only is the average brain sensitivity fairly low (on the order of 20% for typical scalp to brain distances in the frontal region for example), but the measurement has higher sensitivity to scalp than to brain blood flow. Of note, these results assume that the entire autocorrelation decay is being fitted. The early part of the decay is driven by photons that experience more scattering events, and thus it has higher CBF sensitivity; however, limiting the fit to the upper part of $g_{2}$ leads to significant increases in ${\mathrm{BF}}_{i}$ estimate variability and overall lower cerebral perfusion measurement SNR (see Supplementary Material in Ref. 36).

**Fig. 3:**
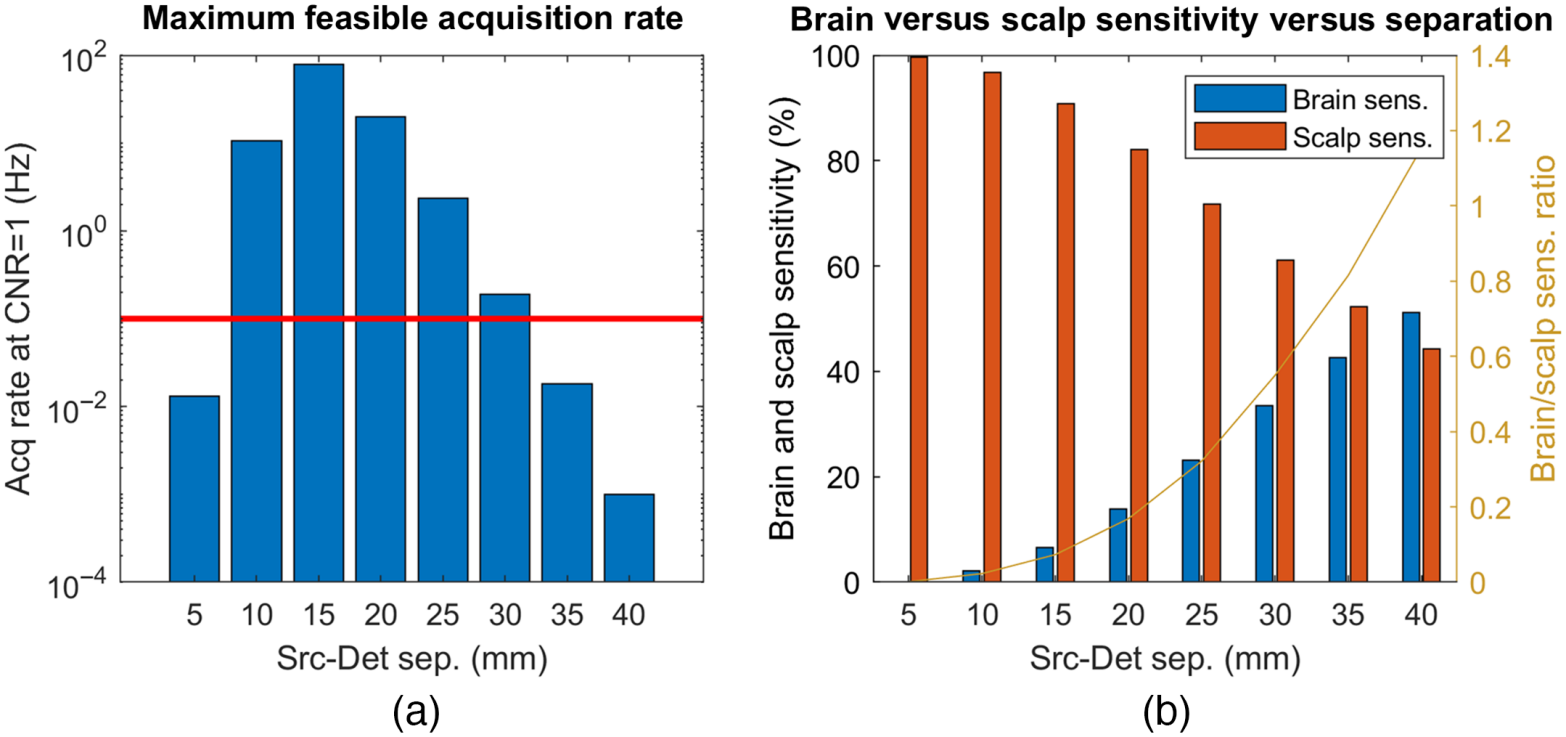
Monte Carlo simulation-driven exploration of DCS measurement noise performance and cerebral versus extracerebral sensitivity: (a) achievable acquisition rates at unitary CNR, for typical DCS measurements at 850 nm across different source-detector separations (horizontal red line drawn at 0.1 Hz) with CNR defined as the fraction of the true cerebral perfusion change recovered by the DCS measurement divided by the standard deviation of the BFi estimate and (b) corresponding brain and scalp flow change fractional sensitivities versus separation.

The noise performance versus cerebral sensitivity trade-off for DCS is in fact perhaps the biggest challenge to the wider adoption of this technology. Figure 3 displays the achievable data acquisition rate for a unitary contrast to noise ratio (CNR) and the relative brain to scalp sensitivity for DCS measurements at a range of source-detector separations between 5 and 40 mm. These results are derived from Monte Carlo simulations on a simplified two layer slab geometry with a 12-mm superficial layer thickness (a somewhat conservative assumption in light of the actual scalp to brain distances shown in Fig. 2), assuming the same optical properties as Ref. 35 and photon count rates typical of our experimental data (11 kcps at 25 mm, scaled across other distances based on light fluence estimations). A step change in CBF was simulated versus baseline conditions, and CNR was defined as the fraction of the true cerebral perfusion change recovered using DCS measurements (under a homogeneous medium assumption) divided by the standard deviation of the ${\mathrm{BF}}_{i}$ estimate.

As seen in Fig. 3(a), the best CNR for CBF monitoring (notwithstanding physiological noise) is actually found at short separations, but at those distances there is little brain sensitivity, especially compared with scalp sensitivity. In contrast, the longest source-detector separation in which measurements are feasible with a reasonable integration time (less than 10 s, shown with a red line in Fig 3(a)) is ∼30  mm. However, even at 30 mm, we remain more sensitive to scalp than brain physiology.

Recently, excitement has been building toward the use of DCS for applications beyond baseline physiology monitoring, specifically for functional brain activation^37^^–^^40^ and to assess the critical closing pressure of cerebral vasculature^41^^–^^43^, a close surrogate of intracranial pressure with substantial clinical significance. However, these applications demand high data acquisition rates to resolve fast flow dynamics, including the detailed pulsatile flow shape.

Active technical development in the DCS field is thus focused on improving measurement SNR and increasing the fidelity of brain and other deep tissue perfusion measurement in conjunction with novel modeling and data processing algorithms, as well as on reducing the cost burden of implementing blood flow monitoring instrumentation.

## 3 Directions of Technical Development

### 3.1 Hardware Approaches for SNR/Depth Sensitivity Improvement

#### 3.1.1 Multi-source and parallel detection DCS

The simplest approach to increasing DCS measurement SNR is increasing the amount of light delivered to the tissue as SNR is directly proportional to the photon counting rate.^44^ The maximum permissible exposure is limited by safety standards (ANSI Z136.1 in the United States); however, two illumination positions separated by ∼5  mm can fit in most DCS probe designs, or simply a large spot (5 mm or larger) can be used, though it potentially makes short separation measurements more difficult. Additionally, multiple photon counting detection channels can be used to sample multiple speckles, as first demonstrated by Dietsche et al.^45^ and further advanced by the availability of SPAD cameras with 1024 or more channels.^46^^,^^47^ However, this approach has a high cost, and the improvement scales only with the square root of the detector channel number.

#### 3.1.2 Heterodyne/interferometric detection

Another major avenue for increasing both the noise performance and the robustness of DCS measurements is the addition of a reference arm to achieve heterodyne interferometric detection, in which some of the source light is recombined with the photons collected from the tissue before the detector. For a standard DCS setup, conversion to heterodyne measurement doubles the SNR of the autocorrelation measurement and increases the SNR of the ${\mathrm{BF}}_{i}$ time course even more, especially at large source-detector separations.^48^ Further, the measurement becomes substantially insensitive to environmental light, a significant advantage for practical use cases. By shifting the measured signal to a high intensity level, another important advantage of heterodyne detection is enabling the use of lower cost, noisier devices, and making it possible to use non-photon counting detectors, such as complementary metal-oxide-semiconductor (CMOS) cameras, as further detailed below. One downside is the stricter stability requirement for the laser source as directly coupled light dominates the detected signal.

#### 3.1.3 Multispeckle camera-based methods

An alternative approach to multispeckle detection that is gaining increasing interest in the field is the use of low(er) cost CMOS cameras as massively parallel detector arrays. This has been demonstrated in the temporal domain, using a high-speed line scan cameras in conjunction with heterodyne detection to sample light collected by a multimode fiber;^49^ in the Fourier domain, using heterodyne holographic demodulation across multiple speckles;^50^ and in the spatial domain, imaging the speckle pattern collected at some distance away from the illumination location both without and with the use of a reference arm (termed speckle contrast optical spectroscopy,^51^ and interferometric speckle visibility spectroscopy,^52^ or multiexposure interferometric diffusing wave spectroscopy,^53^ respectively). These approaches can exceed the performance of standard DCS even without the reference arm and can offer nearly two orders of magnitude improvement in the interferometric version, though the use of multimode detection fibers may increase sensitivity to motion artifacts.

#### 3.1.4 Long pathlength photon selection

To improve sensitivity to flow in deep tissues, a number of methods have been proposed to isolate the photons that travel at depth and reject those that only probe superficial tissues. These include time-of-flight selection (time-resolved/time-domain DCS^54^ and the related iNIRS technique^55^), pathlength selection through coherence gating,^56^ and acoustic (ultrasound) tagging.^57^^,^^58^ A major advantage of these techniques is that large source-detector separations are no longer needed, enabling compact probe design and/or dense spatial sampling and increased resolution, for example, for functional brain imaging. Further, time-domain DCS and iNIRS intrinsically sample the optical properties of the sample as well, providing both spectroscopic and flow property measurement.

#### 3.1.5 Long wavelength operation

As DCS is based on light scattering, it has recently been shown that substantial benefits accrue from operating at longer wavelengths in the water absorption local minimum between 1050 and 1100 nm and in particular at 1064 nm, where there is a wide availability of optoelectronic components, including high power laser sources, which were initially developed for the telecom industry.^59^ Due to increased skin exposure limits, slower autocorrelation decay, lower scattering, and lower energy per photon, an order of magnitude improvement is available in DCS measurement SNR. However, the lack of suitable semiconductor photon counting detectors represents a significant challenge, and initial demonstrations have used superconducting nanowire devices that are cryocooled and hence expensive and noisy.^60^^,^^61^

#### 3.1.6 Summary

DCS and related techniques are the focus of intense technical development activities as described above and summarized in Table 1. Many of these approaches can potentially be combined to compound benefits, and several orders of magnitude improvements in SNR are likely; this can translate to faster acquisition rates and/or the ability to conduct measurements at larger source-detector separations with higher brain sensitivity.

**Table 1:** Summary of technical development avenues for improving DCS SNR and depth sensitivity.

Method	Benefits
Parallel illumination and detection	SNR increase proportional to source power and to the square root of the number of detector channels.
Heterodyne/interferometric detection	Doubling of autocorrelation SNR allows for the use of low-cost, higher noise detectors, and robustness against environmental light conditions.
Multi-speckle camera-based detection	Substantial SNR benefit due to sampling large numbers of speckles, especially in conjunction with heterodyne detection.
Long pathlength photon selection	Increased depth sensitivity, independence of source-detector separation and thus higher spatial resolution for tomography.
Long wavelength operation	Substantial increase in available photon throughput and thus measurement SNR, availability of high power sources.

### 3.2 Advanced light Transport Modeling and Calibration Maneuvers

Inspired by efforts in the NIRS community, advanced multilayer correlation transport models have been developed for DCS, using both analytical^38^^,^^62^^,^^63^ and Monte Carlo simulation-based^64^^,^^65^ approaches. By leveraging the differential depth sensitivity of the different regions of the autocorrelation curve, generally augmented by multidistance measurements, these methods seek to separately estimate superficial versus deep tissue blood flow. Several studies have reported the successful recovery of cerebral perfusion changes in the presence of extracerebral contamination during physiological maneuvers, such as hypercapnia.^36^^,^^66^

A downside of this approach is the increase in estimated ${\mathrm{BF}}_{i}$ time course noise due to limited cerebral perfusion sensitivity. Further, setting the appropriate geometry of the modeled layers can be challenging, even if structural medical imaging scans are available, because the Monte Carlo model fidelity is not sufficient to allow for the direct use of segmented anatomical information.^36^ To aid in the selection of model parameters, the use of pressure modulation maneuvers was pioneered by Mesquita and Baker,^67^^,^^68^ based on the principle that superficial perfusion perturbations should only impact scalp flow estimates, and brain ${\mathrm{BF}}_{i}$ should remain constant during the pressure period if layer thicknesses are chosen appropriately.

## 4 Future Perspective

We are in an exciting time in the development of noninvasive deep tissue perfusion monitoring technology. DCS and related approaches offer substantial promise in becoming a useful tool for both clinical decision making and functional imaging studies. The basic technology is proven, and the great progress being made in advancing measurement SNR, depth sensitivity, and robustness is likely to bear fruit in the near future. To this end, a focused effort is needed to convert the advances outlined in the previous section into reliable, compact, and easy to use instrumentation that can be brought into clinical spaces and operated by nonexperts.

At the crux of these translational efforts remains the need to ensure measurement accuracy, and, especially for clinical translation, the need to make the ${\mathrm{BF}}_{i}$ values interpretable.

Accuracy can be maximized using real-time evaluation criteria at the beginning of a measurement to ensure good brain sensitivity (such as comparing pressure modulation effects at short versus long separations, seeking locations where long channels display higher ${\mathrm{BF}}_{i}$ values than short channels, using any existing CT or MRI scan to plan probe placement, etc.) in conjunction with multilayer modeling to remove superficial physiology contamination—a task made feasible by leveraging hardware advances that increase both measurement SNR and brain sensitivity.

In parallel, there is a need to go beyond trend monitoring, toward being able to provide absolute perfusion values and establishing normative ranges that can be used in medical decision making. Efforts to calibrate ${\mathrm{BF}}_{i}$ are already ongoing in the field,^19^^,^^21^^,^^69^ but it remains necessary to augment these not just with accurate measurement models as described above but also with validation studies to demonstrate that calibrated DCS perfusion values in clinician familiar units of flow/volume (mL of blood/mL of tissue/second) track those from established MRI and CT perfusion quantification methods in humans.

Last, but not least, there is a need for advances to enable the development of wearable, low-cost DCS devices not only to increase the dissemination of the technology but also to enable studies in naturalistic environments, akin to the developments in the fNIRS field.^70^
